# Modified Sleep Apnea Severity Index and Cardiovascular Risk in CPAP‐Intolerant OSA Patients

**DOI:** 10.1002/lary.70494

**Published:** 2026-03-23

**Authors:** Praneet C. Kaki, Jennifer A. Goldfarb, Melissa Xu, Daniel J. Campbell, Nicole Molin, Erin Creighton, Thomas M. Kaffenberger, Maurits Boon, Colin Huntley

**Affiliations:** ^1^ Sidney Kimmel Medical College Thomas Jefferson University Philadelphia Pennsylvania USA; ^2^ Departments of Otolaryngology & Sleep Medicine Thomas Jefferson University Hospital Philadelphia Pennsylvania USA; ^3^ Department of Otolaryngology University of Pittsburgh Pittsburgh Pennsylvania USA; ^4^ Veteran's Affairs Pittsburgh Healthcare System Pittsburgh Pennsylvania USA

**Keywords:** cardiovascular mortality, cardiovascular risk, CPAP intolerance, hypoglossal nerve stimulation, modified sleep apnea severity index, obstructive sleep apnea

## Abstract

**Objective:**

The modified sleep apnea severity index (mSASI) combines patient anatomy, weight, sleep study metrics, and symptoms into a composite OSA index ranging from 1 (least severe) to 3 (most severe). Our study aims to assess the association between the mSASI and cardiovascular (CV) risk factors in patients undergoing sleep surgery.

**Study Design:**

Retrospective cohort review.

**Setting:**

Single‐institution tertiary care center.

**Methods:**

CPAP‐intolerant OSA patients who underwent hypoglossal nerve stimulation, maxillomandibular advancement, or expansion sphincter pharyngoplasty at our institution from 2014 to 2021 were included. Cardiovascular comorbidities and 5‐year Framingham Risk Score (FRS) were assessed at the preoperative visit. Chi‐squared and Wilcoxon rank sum test analyses were performed using R Studio.

**Results:**

Of the 209 patients included, 118 had an mSASI = 1, 71 had an mSASI = 2, and 20 had an mSASI = 3. Patients with an mSASI of 2 or 3 were more likely to have HTN (33% vs 51%, *p* = 0.011). Baseline mSASI (*β* = 4.4, 95% CI 0.04–8.7) and age (*β* = 1.3, 95% CI 1.0–1.6) were independently associated with increased FRS on multivariable linear regression (*p* < 0.05). However, this association did not persist in secondary models excluding constituent components of mSASI and FRS.

**Conclusions:**

The mSASI may offer additional benefits in assessing OSA risk severity based on CV risk factors compared to the AHI alone. However, given that this association was not replicated in the secondary analysis, further research is needed to evaluate the utility beyond its individual factors and traditional metrics alone.

**Level of Evidence:**

4.

## Graphical abstract

The modified sleep apnea severity index (mSASI) combines patient anatomy, weight, sleep study metrics, and symptoms into a composite OSA index ranging from 1 (least severe) to 3 (most severe). Our study found that mSASI correlated better with baseline cardiovascular risk measured by the 5‐year Framingham Risk Score compared to the AHI.

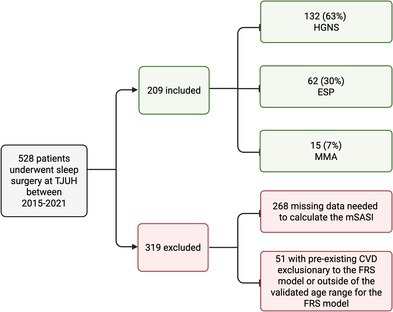

## 1 Introduction

Cardiovascular disease (CVD) remains the leading cause of death in the United States, responsible for 928,741 deaths in 2020 and $407.3 billion in costs between 2018 and 2019 [1]. Obstructive Sleep Apnea (OSA) is a common sleep disorder, present in 23% of women and nearly 50% of men, marked by repetitive airway collapse and intermittent nocturnal airflow obstruction [2]. The oxygen desaturations experienced by patients with OSA have been suggested may impair myocardial contractility, triggering sympathetic nervous system activation, elevating blood pressure, and increasing cardiac stress [3, 4]. Given this association, OSA has been linked to CVD, including myocardial infarction, stroke, coronary artery disease, arrhythmias, and heart failure [3, 5]. Additionally, OSA is thought to accelerate atherosclerosis both as a secondary cause of hypertension and by inducing proatherogenic effects through mechanisms such as systemic inflammation, oxidative stress, and sympathetic nervous system activation [5].

Continuous positive airway pressure (CPAP) therapy can reduce systolic and mean nocturnal diastolic blood pressure, lower stroke‐related mortality observationally, and improve left ventricular function. However, randomized trials report conflicting results on whether CPAP prevents cardiovascular events, potentially due to poor adherence (< 4 h/night), exclusion of symptomatic patients, and use of home sleep apnea testing (HST) instead of polysomnography (PSG) [6, 7, 8, 9, 10]. Thus, CVD risk remains a critical clinical factor in assessing OSA severity.

The apnea–hypopnea index (AHI) is widely used but often criticized for oversimplifying OSA, as it excludes anatomy, disease pathophysiology, and hypoxic burden [11]. The Sleep Apnea Severity Index (SASI), first proposed by Piccirillo et al. in 1998 and modified (mSASI) by Balakrishnan et al. [12, 13]. It combines polysomnographic (AHI and oxyhemoglobin saturation nadir), subjective (daytime sleepiness), and anthropometric (body mass index and tonsil grade) measures into a composite severity staging system scored from 1 to 3, with 3 being the most severe disease burden. The mSASI and SASI showed an improved correlation to patient quality of life and sleep quality questionnaires compared to the AHI within a general sleep medicine clinic population [13]. Furthermore, these measures have been linked to mean arterial pressure (MAP) and C‐reactive protein (CRP), supporting their utility as a comprehensive disease burden measure [13, 14]. A more recent study of a sleep surgery population found higher baseline mSASI to be associated with greater daytime sleepiness following Hypoglossal Nerve Stimulator (HGNS) implant [15].

Baseline OSA metrics that correlate with CVD risk are not well established, although early identification of at‐risk patients is clinically valuable [16]. Although previous studies have suggested the utility of composite measures, such as the mSASI, in correlating with several objective and subjective endpoints in patients with OSA, such studies have not specifically examined whether the mSASI offers superior predictive value for long‐term CVD risk compared to AHI. To help address this gap, we used the validated 5‐year Framingham Risk Score (FRS), which estimates CVD risk based on demographic, clinical, and laboratory data [17]. We selected the 5‐year FRS over the more commonly used 10‐year score to better reflect short‐ to mid‐term cardiovascular risk, to align with our available follow‐up window and offer more timely clinical insights for a surgical OSA population.

In this study, we evaluated whether mSASI more strongly correlates with 5‐year cardiovascular risk than AHI alone in a cohort undergoing sleep surgery. We hypothesized that mSASI would demonstrate stronger predictive value for CVD risk.

## 2 Methods

This study was approved by the Institutional Review Board at Thomas Jefferson University Hospital.

### 2.1 Cohort Selection

**FIGURE 1:**
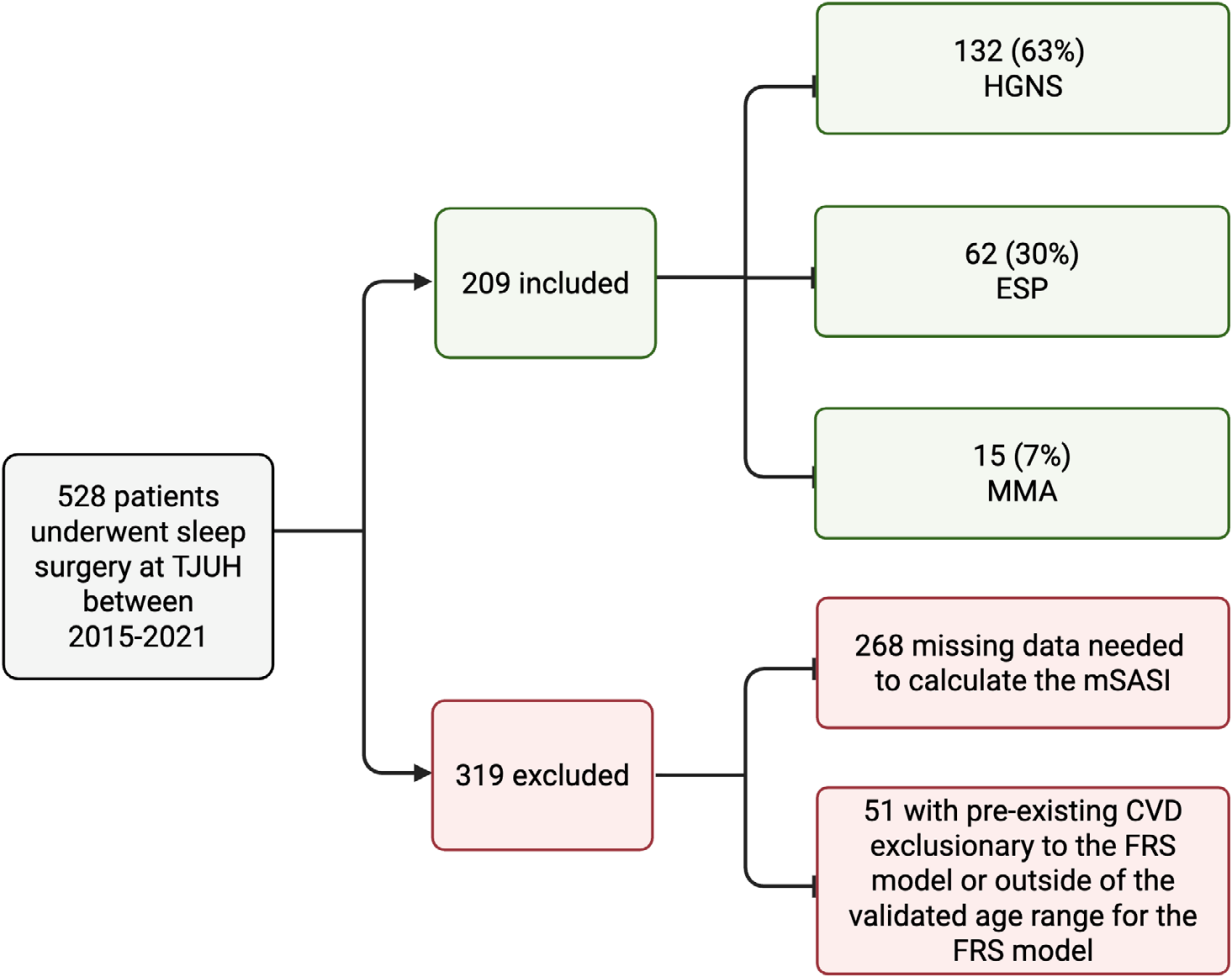
Cohort selection: 528 patients with CPAP‐intolerant OSA underwent HGNS, ESP, or MMA at our tertiary care institution between 2015 and 2021. 268 patients who were missing data for any of the following variables needed to determine pre‐operative mSASI were excluded: BMI, tonsil size, ESS score, AHI, and minimum oxygen saturation. After selecting for patients aged 30–75 without a prior history of CAD, CHF, or CVA/TIA, 209 patients were included in the final cohort, of which 63% of patients underwent HGNS, 30% underwent ESP, and 7% underwent MMA. [Color figure can be viewed in the online issue, which is available at www.laryngoscope.com]

This retrospective cohort study included patients who underwent HGNS (Inspire Medical Systems, Minneapolis, MN, USA), Maxillomandibular Advancement (MMA), or Expansion Sphincter Pharyngoplasty (ESP) for OSA between 2015 and 2021 at a tertiary care facility (Figure 1). All patients had failed CPAP therapy and underwent drug‐induced sleep endoscopy (DISE) preoperatively.

Of 528 patients initially identified, 268 were excluded due to missing data required to calculate the preoperative mSASI (AHI, Epworth Sleepiness Scale [ESS], oxygen saturation [SpO_2_] nadir, BMI, and tonsil size), 37 were excluded for pre‐existing cardiovascular disease incompatible with the FRS model (e.g., MI, CAD, CHF, stroke, or peripheral vascular disease), and 17 were excluded for being outside the FRS‐validated age range (30–74 years) [17]. This resulted in a final cohort of 209 patients (Figure 1) [18, 19].

### 2.2 Variables and Outcome Measures

**FIGURE 2:**
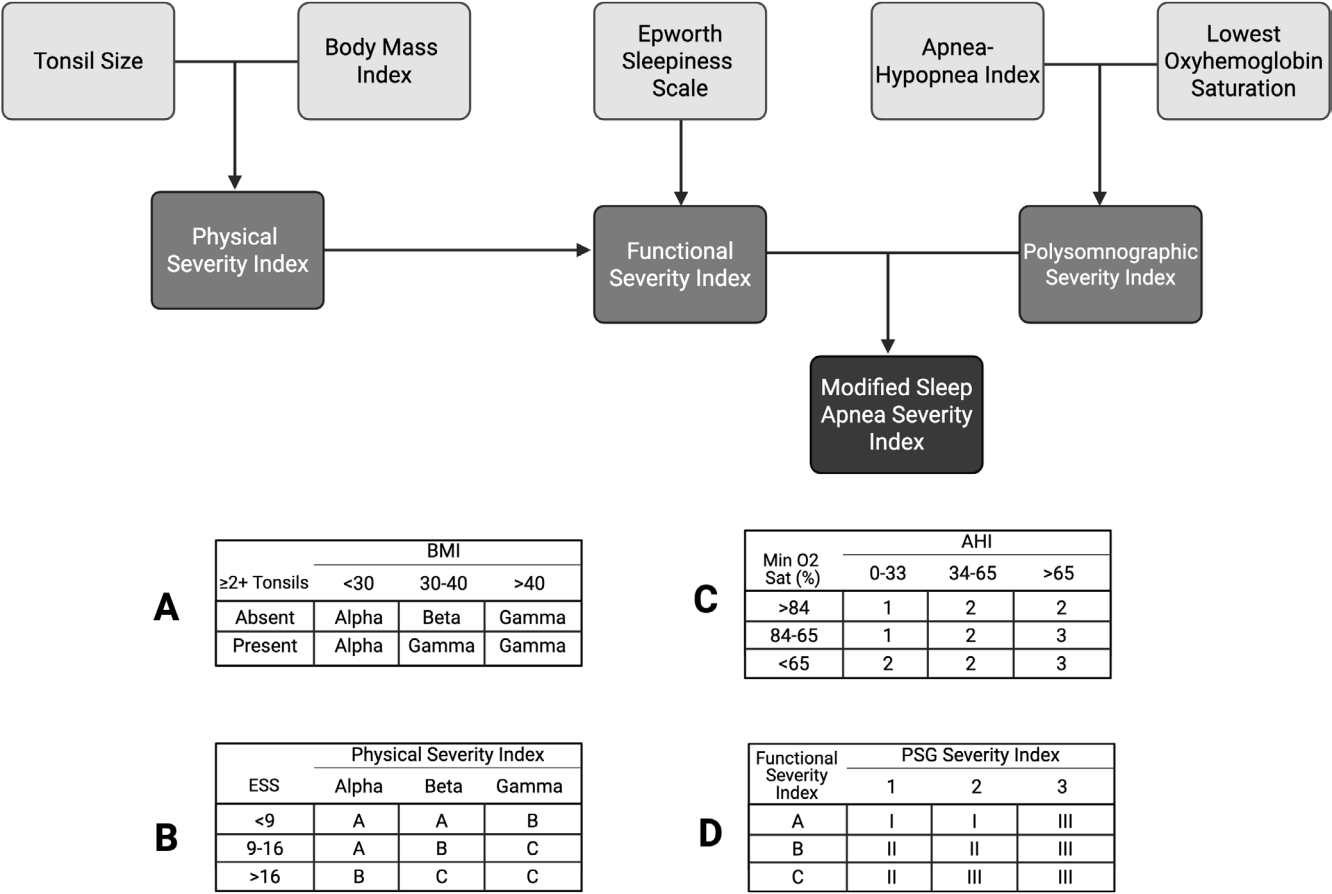
Schematic workflow of the modified sleep apnea severity index (mSASI). Panels A through D illustrate the stepwise integration of physical exam findings, ESS scores, and PSG variables to calculate the mSASI. Panel A: Consolidation of tonsil size and BMI to calculate the composite three‐level physical severity index (alpha, beta, gamma). Panel B: Integration of the physical severity index levels (alpha, beta, gamma) with ESS score categories (< 9, 9–16, > 16) to calculate the functional severity index (A, B, C). Panel C: Consolidation of minimum oxygen saturation and AHI to calculate the three‐level PSG severity index (1–3). Panel D: Final integration of functional severity index levels (A, B, C) and PSG severity index levels (1–3), resulting in a three‐category mSASI (I, II, III). Modified from Piccirillo et al. [12].

Demographics including age, sex, race, and body mass index (BMI) were collected at the time of surgery. Among 209 patients, 141 (67.5%) patients had preoperative PSG study data available to assess OSA. Most studies (90%) defined hypopneas as ≥ 30% airflow reduction lasting ≥ 10 s with ≥ 4% desaturation; the rest used the 3% rule [20]. The remaining studies were full‐night type 3 home tests (eHSTs). AHI (events/h) and SpO_2_ nadir (%) metrics were collected from preoperative sleep studies. OSA severity was defined as: mild (AHI 5–15), moderate (15–30), and severe (> 30 events/h). The preoperative Epworth Sleepiness Scale (ESS) score closest to the time of surgery was reported. The mSASI was calculated by integrating physical exam findings (tonsil size, BMI), symptom severity (ESS), and sleep study metrics (AHI, SpO_2_ nadir) into a composite score from 1 to 3, with higher scores indicating greater OSA severity (Figure 2).

Preoperative CV comorbidities—including type 2 diabetes mellitus (T2DM), atrial fibrillation (Afib), and hypertension (HTN)—were extracted from clinical notes. Total CV comorbidities were defined as the number of these conditions in each patient. The primary endpoint of our study was 5‐year CVD risk per the recalibrated Framingham Risk Score (FRS), which was calculated for each patient through a specific multivariable algorithm that incorporates the following factors obtained at the closest available preoperative timepoint (typically within 8 weeks prior to surgery): sex, age, total cholesterol (mmol/L), HDL (mmol/L), systolic blood pressure (mmHg), on treatment for high blood pressure (yes/no), smoker, and diabetes [17]. Major adverse cardiovascular events (MACE) were also retrospectively tracked through May 2024 and defined as first hospitalization due to MI, stroke, CHF exacerbation, revascularization (PCI or CABG), or all‐cause death [21].

### 2.3 Statistical Analysis

Descriptive statistics were used to characterize the study population and summarize baseline OSA metrics. Continuous variables were reported as mean (standard deviation; [SD]) and analyzed using paired, nonparametric Wilcoxon Rank Sum Testing. Categorical variables were reported as frequency (percentage) and analyzed using Chi‐squared or Fisher's exact tests, as appropriate.

A multivariable regression model adjusting for relevant covariates (age, race, surgical intervention type) and AHI as an important comparator to mSASI were used to identify predictors of Framingham 5‐year CVD risk. Both AHI and mSASI were included in the same model to assess the potentially additional predictive value of the composite mSASI metric over AHI alone; other components of the mSASI were omitted from the primary model. To address potential multicollinearity between the composite mSASI and its constituent individual components, a secondary model specification was performed. In this reduced model, mSASI was evaluated as the primary predictor, while excluding all of its constituent components (e.g., AHI, SpO_2_ nadir, ESS, BMI) and age (a component of the FRS), to reduce collinearity and assess the robustness of its association with the 5‐year FRS. Beta (*β*) coefficients represent the estimated change in the absolute 5‐year FRS or the total CV comorbidities associated with a one‐unit increase in the independent variable (e.g., mSASI). Additionally, a logistic regression model was performed to identify independent predictors of having any CV risk factors. Significance was set at *α* = 0.05. All statistical analyses were performed using R Studio (v2023.03.1).

## 3 Results

### 3.1 Demographics and Baseline Characteristics

209 patients were included in the study (Table 1). The average age across the study cohort was 53.77 years (SD = 10.66). 72% of patients were male and 81% White/Caucasian. Mean preoperative BMI was 29.64 kg/m^2^ (SD = 4.04) and mean ESS was 10.31 (SD = 5.38). Average SpO_2_ nadir was 80.23% (SD = 6.55) and mean AHI was 32.07 (SD = 16.34). OSA severity was classified as severe in 47% (AHI > 30), moderate in 44% (AHI 15–30), and mild in 8.6% (AHI 5–15). Regarding surgical interventions, 63% underwent HGNS, 30% ESP, and 7.2% MMA.

**TABLE 1:** Demographics and baseline characteristics.

Characteristic	*N*	Overall, *N* = 209^a^	1, *N* = 118^a^	2, *N* = 71^a^	3, *N* = 20^a^	*p* ^b^
Age	209	53.77 (10.66)	55.40 (10.88)	51.65 (10.15)	51.65 (9.82)	0.058
Sex	209					0.4
Female		58 (28%)	30 (25%)	20 (28%)	8 (40%)	
Male		151 (72%)	88 (75%)	51 (72%)	12 (60%)	
Race	209					0.10
Non‐White		39 (19%)	18 (15%)	19 (27%)	2 (10%)	
White		170 (81%)	100 (85%)	52 (73%)	18 (90%)	
Surgical intervention	209					**< 0.001**
Expansion sphincter pharyngoplasty		62 (30%)	22 (19%)	32 (45%)	8 (40%)	
Maxillomandibular advancement		15 (7%)	7 (5.9%)	5 (7.0%)	3 (15%)	
Hypoglossal nerve stimulation		132 (63%)	89 (75%)	34 (48%)	9 (45%)	
AHI severity	209					**< 0.001**
Mild OSA		18 (8.6%)	7 (5.9%)	11 (15%)	0 (0%)	
Moderate OSA		93 (44%)	55 (47%)	36 (51%)	2 (10%)	
Severe OSA		98 (47%)	56 (47%)	24 (34%)	18 (90%)	
Baseline AHI	209	32.07 (16.34)	31.05 (12.83)	26.88 (13.94)	56.44 (21.50)	**< 0.001**
Baseline SpO_2_ nadir (%)	208	80.23 (6.55)	80.67 (6.26)	80.35 (5.95)	77.00 (9.40)	0.3
Baseline Epworth Sleepiness Scale	209	10.31 (5.38)	8.56 (4.72)	12.37 (5.50)	13.30 (4.93)	**< 0.001**
Baseline BMI (kg/m^2^)	209	29.64 (4.04)	27.50 (3.09)	32.06 (3.45)	33.63 (2.95)	**< 0.001**

*Note*: Bold indicates statistical significance.

^a^ Mean (SD); *n* (%).

^b^ Kruskal‐Wallis rank sum test; Fisher's exact test.

### 3.2 Cardiovascular Outcomes and Risk Stratified by mSASI and AHI Obstructive Sleep Apnea Severity

Of the 209 patients included, 118 (56%) had an mSASI = 1, 71 (34%) had an mSASI = 2, and 20 (10%) had an mSASI = 3. At least one CV comorbidity was present in 41% of patients with mSASI = 1 and 54% of patients with an mSASI‐2/3 (*p* = 0.058). HTN was more common in patients with mSASI = 2/3 compared to an mSASI = 1 (51% vs. 33%, *p* = 0.011). MACE occurred in 6 (2.9%) patients within 3 years post‐surgery, with no significant difference by mSASI group (4.3% vs. 1.1%, *p* = 0.2; Table 2).

**TABLE 2:** Cardiovascular risk stratified by OSA severity as defined by mSASI.

Characteristic	*N*	Overall, *N* = 209^a^	mSASI 1, *N* = 118^a^	mSASI 2/3, *N* = 91^a^	*p* ^b^
Total number of cardiovascular risk factors	209	0.00 (0.00, 1.00)	0.00 (0.00, 1.00)	0.00 (0.00, 1.00)	0.061
1+ cardiovascular risk factors	209	97 (46%)	48 (41%)	49 (54%)	0.058
Type 2 diabetes mellitus	209	25 (12%)	11 (9.3%)	14 (15%)	0.2
Atrial fibrillation	209	8 (3.8%)	8 (6.8%)	0 (0%)	**0.010**
Hypertension	209	85 (41%)	39 (33%)	46 (51%)	**0.011**
Atherosclerotic Cardiovascular Disease (ASCVD) Risk Calculator Score	42	8.60 (4.32, 17.55)	8.20 (3.90, 12.00)	9.00 (5.90, 18.90)	0.4
Framingham Risk Score (FRS)	134	21.82 (13.28, 39.83)	21.82 (15.63, 39.68)	21.86 (12.01, 39.90)	0.6
Framingham category	134				0.3
High FRS (> 20%)		78 (58%)	44 (59%)	34 (57%)	
Low FRS (< 10%)		20 (15%)	8 (11%)	12 (20%)	
Moderate FRS (10%–20%)		36 (27%)	22 (30%)	14 (23%)	
Major Adverse Cardiac Events (MACE)	205	6 (2.9%)	5 (4.3%)	1 (1.1%)	0.2

*Note*: Bold indicates statistical significance.

^a^ Mean (SD); *n* (%).

^b^ Wilcoxon rank sum test; Pearson's Chi‐squared test; Fisher's exact test.

When stratified by AHI, patients with moderate–severe OSA (AHI > 15) had a similar number of CV risk factors as those with mild OSA (*p* > 0.05). MACE occurred in 0% of mild OSA and 3.2% of moderate–severe OSA patients (*p* > 0.9; Table 3).

**TABLE 3:** Cardiovascular risk stratified by OSA severity as defined by AHI.

Characteristic	*N*	Overall, *N* = 209^a^	Mild OSA, *N* = 18^a^	Moderate/Severe OSA, *N* = 191^a^	*p* ^b^
Total number of CV risk factors	209	0.00 (0.00, 1.00)	0.00 (0.00, 1.00)	0.00 (0.00, 1.00)	0.6
1+ CV risk factors	209	97 (46%)	8 (44%)	89 (47%)	0.9
Type 2 diabetes mellitus	209	25 (12%)	6 (33%)	19 (9.9%)	**0.011**
Atrial fibrillation	209	8 (3.8%)	0 (0%)	8 (4.2%)	> 0.9
HTN	209	85 (41%)	7 (39%)	78 (41%)	0.9
ASCVD risk calculator score	42	8.60 (4.32, 17.55)	7.45 (4.65, 16.95)	8.65 (4.32, 17.55)	0.9
Framingham Risk Score (FRS)	134	21.82 (13.28, 39.83)	20.39 (10.36, 35.20)	21.87 (14.30, 39.80)	0.5
Framingham category	134				0.5
High FRS (> 20%)		78 (58%)	6 (55%)	72 (59%)	
Low FRS (< 10%)		20 (15%)	3 (27%)	17 (14%)	
Moderate FRS (10%–20%)		36 (27%)	2 (18%)	34 (28%)	
Major Adverse Cardiac Events (MACE)	205	6 (2.9%)	0 (0%)	6 (3.2%)	> 0.9

*Note*: Bold indicates statistical significance.

^a^ Mean (SD); *n* (%).

^b^ Wilcoxon rank sum test; Pearson's Chi‐squared test; Fisher's exact test.

### 3.3 Multivariable Regression to Identify Predictors of Total CV Comorbidities and 5‐Year CV Risk

Preoperative mSASI (*β* = 4.4, 95% CI 0.04–8.7, *p* = 0.047) and age (*β* = 1.3, 95% CI 1.0–1.6, *p* < 0.001) were associated with increased FRS on multivariable linear regression adjusting for demographics, preoperative sleep study metrics, and surgical intervention type (Table 4).

**TABLE 4:** Multivariable linear regression models identifying factors associated with 5‐year Framingham Cardiovascular Risk.

Characteristic	Beta	95% CI^a^	*p*
Preoperative mSASI	4.4	0.04, 8.7	**0.047**
Age	1.3	1.0, 1.6	**< 0.001**
Race			
Non‐White	—	—	
White	−5.9	−14, 2.6	0.2
Surgery type			
Expansion sphincter pharyngoplasty	—	—	
Maxillomandibular advancement	−2.8	−16, 10	0.7
Hypoglossal nerve stimulation	0.43	−7.2, 8.0	> 0.9
Baseline AHI	−0.16	−0.34, 0.03	0.091

*Note*: Bold indicates statistical significance.

^a^ CI, confidence interval.

In the secondary model specification excluding constituent variables of mSASI and FRS, pre‐treatment mSASI remained positively associated with FRS, although the effect size was smaller and did not reach statistical significance (*β* = 0.55, 95% CI –4.6 to 5.7, *p* = 0.8; Table S1).

## 4 Discussion

Cardiovascular risk is an important consideration in the management of OSA, which has been linked to HTN, Afib, other arrhythmias, CHF, CAD, CVA/TIA, T2DM, and metabolic syndrome [22]. Despite this relationship, few studies have examined baseline OSA markers that correlate with CV risk—especially in sleep surgery patients. This single‐institution retrospective cohort study found that the mSASI, derived from a combination of polysomnographic, subjective, and anthropometric parameters, was independently associated with 5‐year CV risk as defined by a Framingham CV risk score in patients undergoing surgery for OSA. Specifically, each 1‐point increase in mSASI was associated with a 5.7% increase in 5‐year FRS. In contrast, AHI—despite being the traditional metric for OSA severity—was not associated with increased CV risk after adjustment.

Previous studies assessing the relationship between OSA and CV risk have reported mixed findings. The Wisconsin Sleep Cohort Study published in 2000 found that patients with moderate and severe OSA (AHI > 15) had a 2–3‐fold increase in risk of developing HTN within 4 years compared to healthy individuals [23]. Likewise, a study utilizing a machine learning approach found severe OSA to be associated with greater risk of CV mortality compared to mild OSA [24]. Redline et al. found greater AHI to be associated with stroke risk in men but not women [23]. However, other work found that although AHI predicted CV events on univariate analysis, it lost significance after adjusting for confounders [16]. Notably, metrics such as total sleep time, time under 90% SpO_2_ (T90), and mean HR emerged as more robust predictors [16]. Some studies even suggest an inverse association between OSA severity and CV morbidity, attributed to ischemic preconditioning [25, 26].

Studies have also analyzed the role of hypoxic markers in OSA (e.g., T90, ODI, SpO_2_ nadir, sleep apnea specific hypoxic burden) in predicting CV risk [27, 28, 29]. A large cohort study from the Sleep Heart Health Study identified hypoxia as exerting a mediating effect in the increased risk of CV mortality caused by OSA, although this effect was not significant [30]. Another study by Oldenburg et al. found an independent association between T90, a marker of nocturnal hypoxemic burden, and all‐cause mortality in patients with stable heart failure [31]. Likewise, Baumert et al. found that T90 was an independent predictor of CV mortality, although this was predominantly observed in elderly men [32]. Kendzerska et al. identified T90 as a predictor of composite CV outcomes, although a specific cutoff for stratifying disease severity was not identified, limiting its clinical utility [16]. The controversial impact of CPAP therapy in improving CV risk is reasonable, considering the lack of consensus regarding the role of the AHI and hypoxic burden of OSA on composite outcomes [10, 33]. Of note, most studies prospectively assess fatal (e.g., death from myocardial infarction or stroke) and non‐fatal (e.g., myocardial infarction, non‐fatal stroke, coronary artery bypass surgery, revascularization procedures, new‐onset atrial fibrillation) cardiovascular events. In addition to this, there may be added utility in preoperatively assessing cardiovascular risk using tools such as the Framingham CV risk score, which can serve as a potentially modifiable endpoint to minimize future risk.

At present, most studies focus on individual sleep metrics such as AHI, ODI, and T90, which may not provide a comprehensive measure of OSA disease burden, thereby leading to mixed outcomes regarding OSA severity and treatment on CV risk. AHI is simply a measure of the frequency of disruptions in breathing, ODI characterizes the frequency of desaturations, and T90 is a strict measure of the amount of sleep time spent with SpO_2_ < 90%.

The SASI and mSASI were developed to capture a broader OSA profile by integrating objective, anatomical, and symptomatic data [12, 13, 14]. Our study shows mSASI is an independent predictor of 5‐year CVD risk based on the FRS, even after adjusting for AHI, BMI, ESS, and SpO_2_ nadir [17]. These findings align with prior research showing mSASI correlates better than AHI with patient‐reported quality of life, MAP, and CRP [12, 13, 14]. A prior study found higher AHI values among high‐risk OSA patients per the FRS, although this relationship did not persist after adjusting for confounding factors [34]. We now extend these findings to a CPAP‐intolerant surgical cohort, suggesting mSASI may outperform AHI in predicting CV risk in this population. Older age also predicted higher 5‐year FRS, consistent with the well‐established relationship between aging and CV risk [35, 36].

Our findings are similar to those from a recent study that found a significant positive association between 10‐year FRS and a novel metric called the sleep breathing impairment index (SBII) that is intended to provide a more comprehensive measure of the frequency, duration, and depth of obstructive events than the AHI alone [37]. It is possible that such indices, whether composite measures or novel interpretations of exported polysomnographic data, may better capture the pathophysiologic severity of the OSA disease process compared to traditional single‐variable metrics. In this study, we expanded these findings to a CPAP‐intolerant sleep surgery population, demonstrating that a higher baseline mSASI score is an independent predictor of increased 5‐year CV risk, even after adjusting for traditional sleep metrics such as AHI. In clinical practice, the mSASI may offer improved utility compared to AHI alone in identifying patients at a higher CV risk, which may warrant closer follow‐up to mitigate long‐term complications.

It is important to note, however, that in the secondary model specification excluding components of the FRS and mSASI (AHI, SpO_2_ nadir, BMI, ESS, and age), the association between mSASI and 5‐year FRS did not reach statistical significance. This may reflect limited power due to the modest sample size of patients with complete FRS data (*N* = 134) and suggests that the predictive value of mSASI may be partly driven by its constituent components. These findings highlight the need for future studies with larger cohorts to confirm the independent predictive value of mSASI and further evaluate its clinical utility. Nevertheless, this study still provides important preliminary evidence that mSASI may capture multiple relevant aspects of OSA severity and warrants further investigation as a potential tool for identifying patients at higher cardiovascular risk in a surgical cohort.

There are limitations to this study that should be considered. The retrospective and observational design inherently limits the ability to establish causality and introduces potential biases related to recall bias, data completeness, and selection bias. Furthermore, a number of patients with missing data to calculate the preoperative composite mSASI score were excluded, thus predisposing for a potential selection bias in our patient cohort. Although most patients in our cohort underwent preoperative PSG sleep studies that utilized the 4% desaturation rule to score hypopneas, there is still some heterogeneity across the sleep studies that were referenced to obtain sleep metric data across our cohort because of them being performed at different sleep labs. This heterogeneity may affect the reliability and comparability of AHI and oxygen desaturation values used in our analysis. Despite this limitation, our findings remain relevant as they reflect the diverse and heterogeneous nature of sleep study data encountered in routine clinical practice. Additionally, our study lacks detailed data on the specific reasons for CPAP intolerance—such as mask discomfort, nasal congestion, device‐related anxiety, claustrophobia, or perceived lack of benefit—which limits our ability to interpret the heterogeneity in a real‐world CPAP‐intolerant cohort, reflective of routine clinical practice where not all relevant variables can be systematically captured or controlled. Furthermore, while the FRS is a validated and widely used tool to estimate cardiovascular risk, it remains an indirect surrogate rather than a direct measure of actual cardiovascular events. The relatively low number of recorded MACE during follow‐up—only 7 cases across the entire cohort—limits our ability to draw definitive conclusions regarding real‐world cardiovascular outcomes and prevents meaningful subgroup analysis by mSASI category. As a result, the primary reliance on a predictive risk model (FRS) rather than observed clinical events should be considered when interpreting the strength and clinical significance of our findings. This study only included patients who underwent surgery for uncontrolled OSA following an initial trial of CPAP. This population may therefore represent a more severe phenotype of OSA who is predisposed to greater CV risk compared to a traditional sleep medicine population receiving CPAP therapy alone. Furthermore, while this study aimed to evaluate sleep surgery populations broadly, MMA procedures were relatively underrepresented (7.8%), which may limit the generalizability of our findings among patients undergoing this specific surgical intervention. The lack of long‐term follow‐up and prospective monitoring of patients limits the utility of outcome measures such as MACE. Thus, the FRS, which is an indirect metric calculated at a single timepoint, was utilized as the primary outcome measure.

Moreover, only 11% of our patient population had an mSASI of 3, limiting the statistical power of our analysis, particularly when evaluating the subgroup differences and associations involving the most severe presentations of OSA. Likewise, inherent to the mSASI being limited to a 3‐point scale restricts the amount of variability that can be shown among patients, potentially not accounting for smaller but clinically relevant differences within disease severity. In addition, while the mSASI includes clinically relevant parameters, not all its components—such as tonsil size—are consistently documented or assessed in routine sleep medicine practice, particularly outside surgical contexts. The tool also does not incorporate several important anatomic features of OSA, such as tongue base collapse, or more comprehensive measures of hypoxic burden—beyond SpO_2_ nadir—that may better capture the full spectrum of disease severity.

Future directions include employing a prospective study design to follow patients over time to monitor adverse CV outcomes, expanding this study to non‐surgical OSA patients, and including multiple institutions for a larger, more diverse patient population. Additionally, future research could build upon our results by longitudinally assessing post‐operative mSASI scores at various time points and evaluating their correlation with long‐term CV risk. Ultimately, it remains important to investigate additional measures that comprehensively capture the full breadth of OSA severity to achieve more consistent and reliable conclusions about CV risk.

## 5 Conclusion

This study in patients presenting for sleep surgery found higher baseline mSASI to be an independent predictor of increased 5‐year CV risk per the FRS. However, after adjusting for the individual components of the mSASI index and FRS, the association was no longer statistically significant. These results preliminarily suggest that the mSASI may help identify patients at risk, but its independent prognostic value remains to be fully established. There were no significant differences in the incidence of major adverse cardiovascular events across baseline mSASI cohorts otherwise. It is important to further explore this trend and evaluate other measures of OSA pathophysiological burden to assess the implications on CV outcomes.

## Funding

The authors have nothing to report.

## Conflicts of Interest

C.H.—Research support Inspire and Nyxoah. Consulting Inspire, Nyxoah, Avivomed.

M.B.—Chief Medical Officer for Nyxoah.

T.M.K. reports being a consultant for Inspire Medical and Nyxoah SA; he additionally receives Institutional Research Support from Cryosa Inc. Additionally, he is employed by the Veterans Affairs Medical Center and the content herein does not represent the views of the United States Government.

## Supporting information


**TABLE S1:** Secondary model specification—multivariable linear regression evaluating the association between mSASI and 5‐year Framingham cardiovascular risk, independent of constituent variables (i.e., AHI).
