# Supplementary material for: Modified Sleep Apnea Severity Index and Cardiovascular Risk in CPAP‐Intolerant OSA Patients
Source: Laryngoscope. 2026 Mar 23;136(7):3262–70. doi: 10.1002/lary.70494 (PMC13253168; doi:10.1002/lary.70494)
Supplement: Supplementary file 1 — TABLE S1: Secondary model specification—multivariable linear regression evaluating the association between mSASI and 5‐year Framingham cardiovascular risk, independent of constituent variables (i.e., AHI). [file LARY-136-3262-s001.docx]

**Supplemental Table 1:**  Secondary Model Specification– Multivariable Linear Regression Evaluating the Association Between mSASI and 5-Year Framingham Cardiovascular Risk, Independent of Constituent Variables (ie, AHI)

| **Predictors of 5-year Framingham Cardiovascular Risk Score** | **Beta** | **95% CI***^1^* | **p-value** |
| --- | --- | --- | --- |
| Preoperative mSASI | 0.55 | -4.6, 5.7 | 0.8 |
| Race |  |  |  |
| Non-White | — | — |  |
| White | -9.4 | -20, 0.95 | 0.075 |
| Surgery Type |  |  |  |
| Expansion Sphincter Pharyngoplasty | — | — |  |
| Maxillomandibular Advancement | -0.50 | -17, 16 | >0.9 |
| Hypoglossal Nerve Stimulation | 13 | 3.6, 21 | **0.006** |
| *^1^*CI = Confidence Interval | | | |
